# A Proposed Quantitative Index for Assessing the Potential Contribution of Reprogramming to Cancer Stem Cell Kinetics

**DOI:** 10.1155/2014/249309

**Published:** 2014-05-12

**Authors:** Xuefeng Gao, J. Tyson McDonald, Mamta Naidu, Philip Hahnfeldt, Lynn Hlatky

**Affiliations:** Center of Cancer Systems Biology, GeneSys Research Institute, Tufts University School of Medicine, 736 Cambridge Street, SEMC-CBR1, Boston, MA 02135, USA

## Abstract

Enrichment of cancer stem cells (CSCs) is thought to be responsible for glioblastoma multiforme (GBM) recurrence after radiation therapy. Simulation results from our agent-based cellular automata model reveal that the enrichment of CSCs may result either from an increased symmetric self-renewal division rate of CSCs or a reprogramming of non-stem cancer cells (CCs) to a stem cell state. Based on *plateau-to-peak* ratio of the CSC fraction in the tumor following radiation, a downward trend from peak to subsequent plateau (i.e., a *plateau-to-peak* ratio exceeding 1.0) was found to be inconsistent with increased symmetric division alone and favors instead a strong reprogramming component. The two contributions together are seen to be the product of a dynamic equilibrium between CSCs and CCs that is highly regulated by the kinetics of single cells, including the potential for CCs to reacquire a stem cell state and confer phenotypic plasticity to the population as a whole. We conclude that tumor malignancy can be gauged by a degree of cancer cell plasticity.

## 1. Introduction

Glioblastoma multiforme is the most frequent and most deadly primary brain tumor in adults. A correlation between stem cell-associated marker's expression in GBM and poor prognosis has been observed [1–3]. CSCs in GBM have been shown to be treatment resistant due to their frequent quiescent state, more efficient DNA damage response mechanisms, and microenvironmental survival cues [4–7]. CSC enrichment in GBM has also been found after classic anticancer treatments including radiotherapy [4, 7, 8]. Previously, we and others reported that ionizing radiation (IR) could increase the number of CSCs through advanced DNA-damage repair mechanisms [4, 8], survival and subsequent expansion of the (more resistant) quiescent fraction of CSCs as they return to a proliferative state [9], a switch from asymmetric to symmetric CSC self-renewal division [10–12], and faster cell cycling of CSCs [10, 12]. However, those studies, which were based on the reasonable belief that there is no return path from a non-stem cell to a stem-like state, only partially explained the enrichment of CSCs and the resulting impact on GBM recurrence.

CSCs are thought to be generated from genetic or epigenetic mutations in normal stem cells and progenitors [13]. While the longevity and the functional self-renewal pathways make normal stem cells the most likely cells of origin, recent studies reinforced the fact that differentiated progenies also retain significant developmental plasticity and can be induced to become tumorigenic by a wide variety of experimental approaches [14–18]. Indeed, CD133^−^ glioma cells have been shown to form tumors in nude rats and give rise to CD133^+^ cells* in vivo* but not* in vitro* [19], which suggests a certain degree of phenotypic plasticity exists in glioblastoma cells that is highly regulated by host microenvironment. The phenotypic plasticity of a brain tumor progenitor cell has also been observed in oligodendrocyte precursor cells, which can be reprogrammed by extracellular signaling molecules into neural stem cells that then develop into astrocytes, oligodendrocytes, and neurons [20]. More recently, Verma and colleagues showed that defined oncogenic alterations can cause neural stem cells, astrocytes, and differentiated neurons in the central nervous system to undergo dedifferentiation to generate a neuronal stem-like or progenitor state that initiates and maintains the tumor progression as well as to give rise to the heterogeneous populations observed in malignant glioma [14]. The generated tumors resembled the mesenchymal subtype observed clinically in GBM. Collectively, these findings suggest phenotype reversions may occur more extensively than previously thought.

Cellular plasticity has inspired some interest in investigating the reprograming of differentiated cells into a stem-like state, which has occasionally been observed following irradiation [21, 22]. Based on our previous studies and current evidence of cancer cell stemness, we investigated the potential effects of radiation on dictating cell fate decision in GBM, in particular, cellular reprogramming. The present study further supports the proposition that IR-induced modulation of self-renewal kinetics and plasticity in GBM might contribute to tumor recurrence. Implications for the degree to which this may occur in association with tumorigenic potential in a CSC model are discussed.

## 2. Materials and Methods

An agent-based cellular automata model is used to describe the behaviors of individual cancer cells dependent on intrinsic mechanisms of migration, proliferation, and death. The domain is defined as a two-dimensional lattice (*L* × *L* lattice points) under periodic boundary conditions, using a Moore neighborhood with a radius of 1 (i.e., 8 nearest neighbors). At any time a lattice point can contain one single cell or be empty. If a free lattice site is found within the Moore neighborhood of a cell, the maturity of the cell increases, and it can migrate with a probability *p*_*m*_, or divide to produce a new cell provided the maturation (i.e., one cell cycle time *T*_*c*_) has been reached. A proliferative cell turns quiescent when it is completely surrounded by other cells and can reenter the cell cycle when a neighboring free space is available.

Cancer stem and non-stem cells are considered. As per the stem cell hypothesis, cancer stem cells (CSCs) reside at the top of the hierarchy and give rise to progenitor cells, which in turn give rise to non-stem cancer cells (CCs). While CSCs can duplicate for an indefinite amount of time, CCs are able to divide only a limited number of times (c.f., Hayflick limit; [23]). CCs have a probability of acquiring a stem-state through reprogramming. This fact is useful in modeling to track CCs based on the number of divisions *ρ* they have remaining, before finally exhausting their proliferative capacity (assumed to occur after *ρ*_max⁡_ divisions). At that point, the cells become senescent and die when they attempt to divide again [24]. A CSC can give rise to two new CSCs with probability *p*_2_ via symmetric self-renewal division, two CCs with probability *p*_0_ via symmetric differentiative division, or with probability *p*_1_ = 1 − *p*_0_ − *p*_2_ give rise to a CSC and a CC via asymmetrical division. In normal tissues, stem cells are regulated by their microenvironment to achieve an optimal balance between activation, self-renewal, and differentiation. A constant stem cell population implies that *p*_2_ = 0 [25], while in tumors we presume *p*_2_ > 0. Two CCs are generated by duplication of a nonsenescent CC (*ρ* > 0). Nonsenescent CCs have a probability *p*_*r*_ per day of acquiring a stem-state through reprogramming.

After exposure of the model system to radiation, cells become arrested in the cell cycle and attempt to repair radiation-induced DNA damage [26]. We assume that a cell becomes arrested in its cell cycle for a period of time *T*_*a*_ immediately following irradiation. Cell survival probability (SF) after a single dose of radiation is modeled using the established linear-quadratic (LQ) model:

(1) $$\begin{array}{c} \text{SF}=e^{-\lambda \xi (\alpha d+\beta d^{2})}, \end{array}$$

where *d* is the dose and *α* and *β* are cell-specific radiosensitivity coefficients [27]. The *αd* term describes a single-track lethal event and *βd* ^2^ accounts for cell killing after a combination of two independent, potentially repairable events. We introduce *λ* as a radiation protection factor for quiescent state and *ξ* as radiation protection factor for CSCs [12].

### 2.1. Parameterization

Model parameters with their values are summarized in [12], most of which are based on recent experimental data using the U87-MG glioblastoma cell line. The size of the 2D domain is given by *L* = 5000. With an average U87-MG cell diameter of 10 *μ*m, one lattice point is therefore 100 *μ*m^2^ in size. Using the 8-cell Moore neighborhood, the estimated cell displacement in a migration step is 10.7 *μ*m [28]. Given the initial migration frequency *p*_*m*_ = 0.3, the average* in silico* cell migration speed was measured as 6.9 *μ*m per simulation step. With the U87-MG cells moving 23.4 *μ*m per hour* in vitro*, one hour was defined approximately as 4 simulation steps. The average cell cycle time of U87-MG cells was estimated to be *T*_*c*_ = 25 hours [12].

Hillen et al. have shown that CSC-driven tumor growth models are naturally equivalent which are independent of the frequencies of symmetric differentiative division and asymmetric division [29]. Therefore we assumed *p*_0_ = 0 in the present model. The average self-renewing symmetric division probability *p*_2_ = 0.35–0.45 was estimated [12] by comparing the frequency of CSCs in the simulated tumors with the fraction of CD133^+^ cells in the U87-MG cell line (c.f., %CD133^+^ = 1.8% reported in Kim et al. [8]). We assigned a value for the division capacity *ρ*_max⁡_ = 10 of non-stem cells based on previous simulation results that revealed aggressive tumor progression at that value [30]. Based on the facts that CSCs exist in a metastable state, and the flux of CC into the CSC is relatively low or even nonexistent under most conditions [31], we assumed there was no intrinsic interconversion reprogramming in the control population, that is, *p*_*r*_ = 0 as a default condition.

Radiation-induced cell cycle arrest of U87-MG cells was observable through a decreased mitotic index immediately after irradiation that returned to control levels after about 16 hours [26, 32]. Accordingly, we assigned a cell cycle arrest time *T*_*a*_ ∈ [0, 16  hours] for each cell from a uniform distribution. Using clonogenic assays for long-term survival of U87-MG cells after single doses of radiation ranging from 0 to 16 Gy, the unconstrained best-fit values of radiosensitivity coefficients *α*, *β* in the LQ model (1) were derived as 0.3859 and 0.01148, respectively. By applying half radiosensitivity for non-cycling cell state [33, 34], we assumed *ξ* = 1 for proliferative cells, and *ξ* = 0.5 for quiescent cells. A higher radioresistance of stem state has been demonstrated in previous study [4] where we derived *λ* = 0.1376 for CSCs and *λ* = 1 for CCs [12].

## 3. Results 

### 3.1. Radiation-Induced Reprogramming May Contribute to Enrichment of CSCs

**Figure 1:**
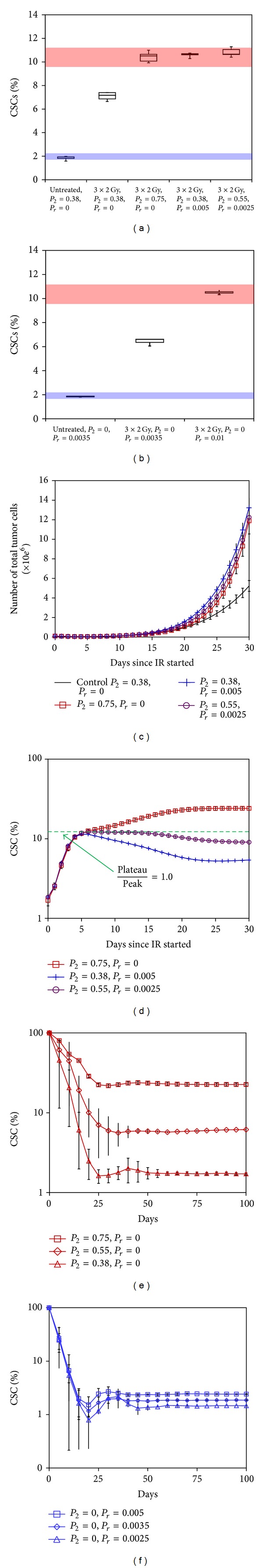
The bottom and top transparent regions in (a) and (b) represent the percentages of CD133^+^ subpopulation in the U87-MG cell line before and after fractionated irradiation (3 × 2 Gy), respectively [8]. (a) CSC fraction* in silico* after fractionated irradiation (3 × 2 Gy) under different mechanisms (means ± SD, *n* = 5). (b) The CD133^+^ fraction observed in the U87-MG population before and after fractionated irradiation can also be reproduced* in silico* by applying an induced reprogramming rate.* In silico* tumor regrowth dynamics for different rates of symmetric divisions and reprogramming: (c) total tumor cell number and (d) percentage of CSCs. Tumors initiated by a single surviving CSC maintain a steady proportion of CSCs under either (e) a constant symmetric division rate or (f) a constant cell reprogramming rate.

By monitoring the tumor population dynamics over time, we recorded the number of both phenotypes every 24 hours for 30 days after start of radiation treatment. At 48 hours after treatment with 3 × 2 Gy, we compared the average percentage of CSCs within individual tumors to the experimental data in literature [4, 8]. Our previous study has shown that the higher fraction of CD133^+^ cells observed after 3 × 2 Gy IR (c.f., %CD133^+^ = 10.5% [8]; Figure 1(a)) could not be recapitulated in our* in silico* study assuming the same cell kinetics as in sham irradiated control (c.f., %CSCs = 7.09%, *p*_2_ = 0.38; Figure 1(a)), and the larger size CSC pool may arise from a IR-induced substantial increase in self-renewal divisions (%CSCs = 10.43%, *p*_2_ = 0.75; Figure 1(a)). [12]. Instead, in present model, by introducing just 0.5% (*p*_*r*_ = 0.005, Figure 1(a)) reprogramming probability for CCs after the second fraction of radiation in a 3 × 2 Gy course, 10.6% of CSCs were observed in the tumor, a value also close to the reported CD133^+^ ratio* in vivo* [8]. Similarly, a good fit was also observed for a combination of partial modulation of both mechanisms (%CSC = 10.8%, *p*_2_ = 0.55, *p*_*r*_ = 0.0025; Figure 1(a)).

If the induced higher symmetric self-renewal divisions or cell reprogramming is inheritable, aggressive tumor regrowth is observed (Figure 1(c)). Importantly, if the increased symmetric self-renewal division rate of CSCs is persistent, the ratio of CSCs in recurrent tumors is seen to peak around day 5 after IR (Figure 1(d)), even if reprogramming is assumed to take place, whereas an increase in the ratio of CSCs is observed for an extended period of time in recurrent tumors if a higher symmetric self-renewal division rate is induced (*p*_2_ = 0.75, “□” curve; Figure 1(d)). By drawing a line of* plateau-to-peak* = 1.0 (green dashed line; Figure 1(d)), the trend of this ratio can roughly indicate that the dominated driving force behind the enrichment of CSCs after radiation treatment might be cell reprogramming if the* plateau-to-peak* ratio drops; otherwise we can infer an increase in symmetric self-renewal CSC division rate as a motivating force.

### 3.2. Dynamic Equilibrium between CSCs and CCs Is Regulated by Single-Cell Kinetics: Symmetric Self-Renewal Division and Cell Reprogramming under Both Normal and Irradiation Conditions

Our simulations further suggest, as expected, that starting from a single CSC, the dynamic equilibrium between CSCs and CCs would eventually be attained in the progeny population (Figure 1(e)). This observation is similar as what Boman et al. demonstrated in their study [35] that a relatively constant stem/non-total cell population ratio is maintained during colorectal cancer development by symmetric stem cell divisions.

Apart from symmetric self-renewal divisions, we find that phenotypic equilibrium also presents under a given rate of cell reprogramming (i.e., *p*_2_ = 0, *p*_*r*_ > 0) and that there is a positive correlation between the cell reprogramming rate and the proportion of CSCs (Figure 1(f)). Since a quantitative measurement of symmetric self-renewal division frequency in GBM cell lines or primary tumors is still to be determined, it is reasonable to investigate another tumor growth scenario wherein no symmetric self-renewal division of CSCs is involved. In our simulations, by assuming an absence of CSC symmetric self-renewal division (i.e., *p*_2_ = 0), and applying a 0.35% of reprogramming rate (i.e., *p*_*r*_ = 0.0035), we could also reproduce the fraction of CD133^+^ cells in the U87-MG cell line (c.f., %CD133^+^ = 1.8% [8]; Figure 1(b)). After the 3 × 2 Gy IR exposure, the enrichment of CSCs could also be reproduced by increasing reprogramming rate from 0.35% to 1% (Figure 1(b)). This finding is in agreement with a study by Gupta et al. who demonstrated that phenotypic equilibrium in populations of breast cancer cells could be achieved via stochastic state transitions [36].

In the irradiated tumors, the intrinsic homeostasis between CSCs and CCs was perturbed by radiation; however, a new phenotypic equilibrium was established on about 25 days after radiation starts (Figure 1(d)). Currently there is no data on GBM studies to validate this prediction; however, the phenotypic equilibrium after irradiation was observed in some other cancer cell lines. For instance, Yang et al. have demonstrated that the homeostasis between CSCs (CD133^+^) and CCs (CD133^−^) in the Swan620 colon cancer cells was disturbed by radiation (gamma-ray, 2 Gy, 6 Gy, or 2 Gy × 3), which reached a final dynamic equilibrium about 18 days after irradiation [37].

In summary, both symmetric self-renewal division and cell reprogramming can independently control the equilibrium proportions of CSCs and CCs within a tumor. However, there is presently no data to determine which of the estimated set of parameters best fits the actual data. Apparently the best solution is to accurately measure the symmetric division rate of CSCs or the reprogramming frequency of the bulk cells in this cell line, which will be part of our future work.

## 4. Discussions 

**Figure 2:**
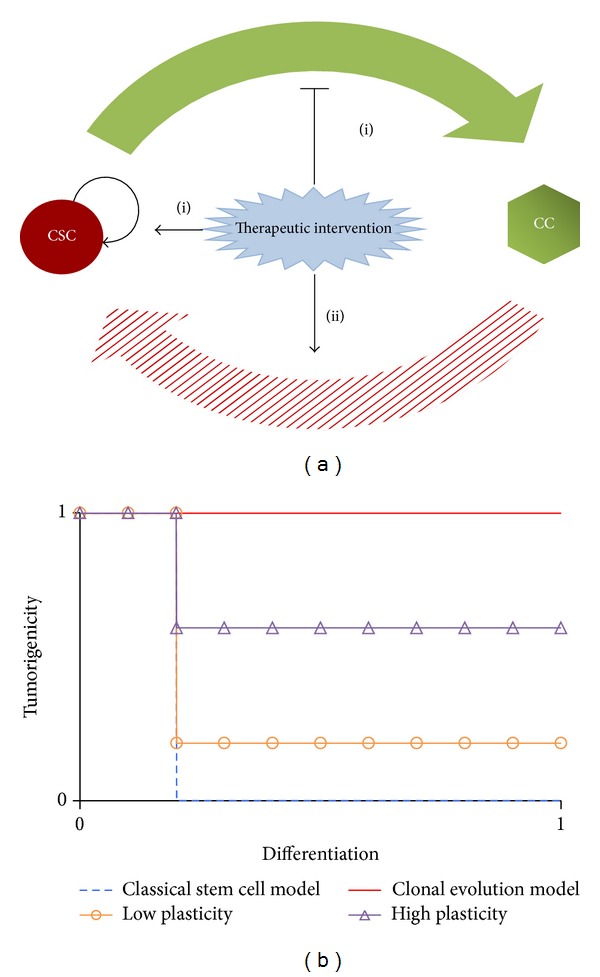
(a) Potential mechanisms by which (fractionated) IR increases the proportion of CSCs. Radiation induces the activation and stabilization of stemness-associated signaling (i) promotes symmetric self-renewal divisions, while repressing differentiation commitment, and/or (ii) evokes reprogramming of progenitor cells into a stem-like state. (b) The tumorigenicity is regulated by the degree of cell plasticity. 0 and 1 stand for minimum and maximum, respectively, in the according axis.

Several studies have shown that IR expands the fraction of cells positive for a CSC maker or side population in both established glioma cell lines and GBM patients [4–7]. Elucidating the mechanisms underlying the enlargement of the CSC pool would be invaluable for informing tumor growth, recurrence, and response to therapies. We previously found that in addition to a higher radioresistance, some CSC self-renewal was required to explain the reported CSC fraction following a certain fractionated IR regimen [12]. However, emerging evidence shows that CSCs may not represent a stable cell type and that the stem-like state may arise in differentiated tumor cells under certain conditions [14–18]. A dynamic equilibrium between CSCs and CCs may therefore exist within tumors that can be shifted bidirectionally by intrinsic or extrinsic signals that influence the probability of interconversion between the CSC and non-CSC compartments [36, 37]. We propose that radiation treatment may induce stemness-associated signaling activation and stabilization, which in turn expand the pool of stem-like cells in GBM through (i) promoting symmetric self-renewal divisions, while repressing differentiation commitment, and/or (ii) evoking reprogramming of progenitor cells into a stem-like state (Figure 2(a)). Indeed, different types of therapy-induced injury to tumor tissue most likely stimulate similar mechanisms to protect and recover the tumor cell population [38]. From a therapeutic point of view, both mechanisms are relevant to therapy-resistance, as CSCs are more resistant to therapies. The* plateau-to-peak* ratio of CSCs might be helpful to identify and distinguish the mechanisms that contribute to CSC enrichment and the consequent high potential of tumor recurrence and suggest targets for decreasing recurrence, for example, the self-renewal capacity of CSCs per se versus the reprogramming capability of the bulk population.

Although great insights have been made using established cancer cell lines, for example, U87-MG, these models have limitations in representing the cellular heterogeneity in tumors. Some parameters and their reported ranges can affect the model result in different extent. For instance, we previously proposed that the accelerated CSC cycling and their increased symmetric division frequency after fractionated irradiation might collaborate on expanding CSC pool [12]. In fact, the cell cycle duration varies largely in some primary GBM tumors (e.g., 75.6 ± 45.7 hours for 24 glioma surgical specimens [39]) and is longer than some known GBM cell lines (e.g., 22 hours for U251-MG [40], 25 hours for U87-MG [12]). Since the frequency of CSCs was usually measured relatively short after radiation exposure (e.g., 48 hours after radiation in [4, 8]), it is reasonable to assume less or no cell proliferation in some irradiated primary GBM before performing CSC frequency measurement. In this scenario, CSC enrichment is more likely driven by cell reprogramming due to the dependence of symmetric CSC divisions on proliferation activities for expanding CSC pool. In our previous study, we proposed that accelerated stem cell cycling after radiation exposure could be another mechanism enriching CSCs [12]. Taken together, our study further suggests that cell cycle analysis can act as another index for estimating the contribution of CSC self-renewing activities in treatment-induced CSC enrichment.

In the future, we will test this model on the clinically relevant subtype of GBM (e.g., mesenchymal, proneural, and proliferative [41]) with some key cellular parameters (e.g., migration speed, proliferation rate, apoptosis rate, life span, etc.) and their reported value range. Ideally, we may have more convincing conclusions about how sensitive this model is to changes in each of the parameters and to what extend this model can generate reliable predictions for different GBM subtypes.

A fundamental question in cancer biology is whether cells with tumorigenic potential are common or rare within tumors. In the clonal evolution model, most cancer cells have tumorigenic potential and intratumoral heterogeneity arises from stochastic genetic and/or epigenetic changes. Tumorigenic potential is not necessarily affected by cell differentiation or phenotype changes [42] (Figure 2(b), solid line). Evidence regarding CSCs to date tends to support the theory that only a minor subset, rather than the bulk population, has tumorigenic potential. A tumor is hierarchically organized, with differentiation of CSCs giving rise to a heterogeneous feature, with the resulting non-stem cancer cell component demonstrating a loss of tumorigenic potential (Figure 2(b), dashed line). When cell plasticity is involved in the CSC model, nontumorigenic cells can revert to higher levels of the hierarchy, gradually or instantly reacquiring a stem-like state. In this case, the reprogramming frequency and the underlying degree of cell plasticity become a determining feature in tumor progression potential (Figure 2(b), “Δ” and “∘” lines). Thus, tumors with high stemness could be derived by high plasticity of the cells, independent of their self-renewal ability, increasing the CSC fraction and overall tumorigenicity. Importantly, this finding could also explain why the frequency of CSCs is highly variable between tumors. In addition to regaining stemness, another example of plasticity is seen in the ability of GBM cells to acquire endothelial-like properties (e.g., via trans-differentiation), including their alignment to form pseudovascular structures, which participate in processes of neovascularization and the formation of a fluid-conducting, matrix-rich meshwork [43]. However, it is important to note that, while a higher reprogramming rate may cause a higher frequency of CSCs in a tumor, it is not appropriate to predict malignant potential outside the context of the tumor microenvironment and the host system. Additionally, another key question that has yet to be answered is whether these radiation-evoked mechanisms are transient or persistent.

Cell plasticity raises a huge challenge to the already complicated CSC research. So far, spontaneous reprogramming has only been widely observed in normal tissues, which suggests the mechanism of reprogramming is highly regulated to maintain the normal tissue homeostasis. However, if cancer cells are commonly out of regulation and have relatively higher plasticity, conventional therapies may have to be revisited to check if the introduced disturbances have the potential to modulate cell plasticity. In addition to the identification of CSCs, we need to investigate how CSCs and differentiated bulk tumor cells dynamically respond to microenvironmental changes [44]. For example, hypoxia (HIF1*α*) [45, 46], epithelial-mesenchymal transition [47], inflammatory cytokines (e.g., IL-6 and TGF*β*) [48, 49], and embryonic microenvironments [50] can all promote the reprogramming of CCs and increase the overall stemness of the tumor. It is worth restating that understanding what controls the maintenance of the stem-like and differentiation states may give insights into the cellular signals involved in cancer and may ultimately lead to the development of more efficient anticancer therapies.

## 5. Conclusions

Through quantitative analysis of cellular plasticity in the context of tumorigenic potential, our study concludes the following.

- Both an increase in symmetric self-renewal division rate and cell phenotype reprograming might contribute to enrichment of CSCs and to GBM recurrence following radiation treatment. Importantly, these two mechanisms might be distinguished from the* plateau-to-peak* ratio of the CSC fraction when cell proliferation presents within certain time after irradiation.
- A dynamic equilibrium between CSCs and CCs can be established by either reprogramming or symmetric self-renewal division under normal and irradiation conditions.
- Cell reprogramming can be an essential part of the tumorigenesis process. The degree of cancer cell plasticity may be a crucial property that adjusts overall tumor stemness and promotes malignancy.
- It will be necessary to characterize the quantitative change of cancer cell plasticity in response to therapeutic intervention in order to inform the objective of CSC control presumed to be necessary to accomplish tumor suppression.
